# Characterisation of the wound microbiome and antimicrobial resistance profiles in clinical isolates from epidermolysis bullosa patients

**DOI:** 10.1186/s13023-026-04295-5

**Published:** 2026-03-07

**Authors:** Anteneh Amsalu, Hanif Haidari, Bianca Mirco, Victoria Rudolph-Stringer, Sophie Walter, Anna Antipov, Dédée F. Murrell, Zlatko Kopecki

**Affiliations:** 1 Future Industries Institute, University of South Australia, Mawson Lakes, SA 5095 Australia; 2 College of Medicine and Public Health, Flinders University, Bedford Park, SA 5042 Australia; 3 Department of Dermatology, St. George Hospital, Sydney, Australia; 4 Faculty of medicine, University of New South Wales, Sydney, Australia

**Keywords:** Antimicrobial resistance, Blistered skin, Chronic wound, Epidermolysis bullosa, Wound microbiome, *Staphylococcus aureus*, 16S rRNA sequencing

## Abstract

**Introduction:**

Epidermolysis bullosa (EB) encompasses a rare, inherited group of skin disorders characterised by skin fragility, leading to painful, chronic wounds. While recent international studies have reported reduced microbial diversity and altered bacterial composition in EB blistered skins, no microbiome or antimicrobial resistance data is available for Australian EB patients. The objectives of this study were to characterise the wound microbiome of Australian EB patients and identify bacterial pathogens and their associated antimicrobial resistance profiles.

**Methods:**

A total of 15 chronic wound swabs were collected from 10 EB patients. Bacterial identification was performed using standard culture methods and MALDI-TOF; for a selected resistant subset, whole-genome sequencing (WGS) was conducted on two strains, and full-length 16S rRNA gene sequencing was performed on 10 swab samples. Antimicrobial susceptibility profiles were determined using disk diffusion assays.

**Result:**

PacBio Full-length 16S rRNA sequence analysis revealed a high relative abundance of the genera *Staphylococcus* and *Streptococcus*, with *Staphylococcus aureus* being the most frequently detected species. Among the 27 cultured bacterial isolates, 81.5% exhibited resistance to at least one antibiotic, and 22.2% were classified as multidrug resistance (MDR). WGS of two selected resistant strains harboured *blaZ*, *mecA*, *ermC* and *mupA* genes conferring resistance to penicillins, cefoxitin, clindamycin and high-level mupirocin, respectively.

**Conclusions:**

This is the first study to profile the wound microbiome and antimicrobial resistance in Australian EB patients. The findings provide the descriptive microbial profiles and resistance patterns, with implications for clinical management of patients in a specific healthcare setting.

**Supplementary Information:**

The online version contains supplementary material available at 10.1186/s13023-026-04295-5.

## Introduction

Epidermolysis bullosa (EB) is a rare group of inherited skin disorders marked by skin fragility, consistent blistering and chronic non-healing wounds [1, 2]. These conditions significantly impact patients quality of life, causing persistent pain, blistering and increased vulnerability to infections [3]. Globally, EB is estimated to affect approximately 500,000 individuals [4]. In Australia, the prevalence is estimated at 10.3 cases per million, with over 1,200 individuals currently living with the condition [5]. EB is classified into four main subtypes: EB simplex (EBS), junctional EB (JEB), dystrophic EB (DEB) and Kindler EB, based on the level of skin cleavage and underlying protein defects [6]. Among these, recessive dystrophic EB (RDEB) represents the most severe subtype [7] and is associated with extensive chronic non-healing wounds, scarring and life-threatening complications such as aggressive cutaneous squamous cell carcinomas (SCCs) [2, 5, 8, 9].

Chronic EB wounds are highly susceptible to microbial colonisation and infection. Complete eradication of microbial colonisation is rarely achievable in EB patients; therefore, the clinical focus lies in controlling pathogenic burden and preventing progression to systemic infection including bacteraemia and sepsis [10–14]. International studies have shown that EB wounds are frequently colonised by potentially pathogenic organisms including *Staphylococcus aureus*, *Pseudomonas aeruginosa*, *Corynebacterium spp.*,* Streptococcus spp.*,* Proteus spp.* [10, 12, 15–18], with microbial composition influenced by EB subtype, wound chronicity, anatomical sites, treatment practices and geographical location [19–21].

Microbiome studies from Chile and Austria, the Netherlands, Japan and Israel have consistently demonstrated dysbiosis and reduced microbial diversity in RDEB wounds, often dominated by *Staphylococcus* species [18, 21–24]. High rates of methicillin-resistant *S. aureus* (MRSA) [14, 15, 17, 23] and resistance to commonly used antibiotics have been reported internationally [10, 15, 17, 19]. The chronic nature of EB wounds, combined with frequent antibiotic use, raises significant concerns about the development and spread of antimicrobial resistance (AMR) due to selective pressure [25]. To date, however, the wound microbiome of Australian EB patients has not been characterised.

Therefore, this study aimed to characterise, for the first time, the wound microbiome and AMR profiles in ten Australian EB patients using a combination of full-length 16S-rRNA sequencing and culture-based methods. This work provides valuable descriptive data on microbial composition and resistance patterns of EB wound isolates in Australian clinical context.

## Materials and methods

### Study design

A cross-sectional study was conducted between August 2023 and December 2024, in which 10 patients with EB were prospectively recruited by a dermatologist. The study was undertaken following ethics approval from the University of South Australia Human Research Ethics Committee (HREC application number: 205664) and Bulberry Ltd. Human Research Ethics Committee (HREC application number: 2022-11-1244-PRE-1). Written informed consent was obtained from all patients on the day of examination, prior to swab collection following the declaration of Helsinki ethical principles for medical research involving human subjects.

### Wound swab collection, transport, and preparation

A total of 15 wound swabs were collected from 10 Australian EB patients by the EB nurse during routine follow-up as part of standard care. Wounds selected for swabbing by the treating dermatologist showed features that raised concern for possible infection or impaired healing, including one or more of the following: increased exudate, malodour, fibrin or slough, erythema, delayed healing, or clinician concern based on wound appearance [26]. For patients with multiple wounds, swabs were taken from each affected site using Levine technique [27]. Three swabs were collected sequentially per wound for routine local clinical microbiology testing, microbiology analysis at the Future Industries Institute, University of South Australia and full-length 16S rRNA sequencing. Most wound swabs were collected from the lower extremities (14/15, 93.3%). One patient with the dominant DEB subtype had developed squamous cell carcinoma (SCC) and one patient had been hospitalized two months prior sampling and was receiving antibiotics at the time of collection. Following collection, swabs were placed into Amies transport medium (Copan, TransSystem, Italy), stored at -20 °C and transported on dry ice to the Microbiology Laboratory at Future Industries Institute, University of South Australia.

### Isolation and identification of bacteria from wound swab

A swab from Aimes transport medium was seeded on McConkey agar, 5% horse blood supplemented Colombia agar, and mannitol salt agar. The plates were then incubated at 37 °C for 24 h and plates without growth were further incubated for up to 48 h. Colonies were differentiated by colour, form, haemolysis, consistency and purified by subculturing on the same plate. Following colony purification, morphologically distinct colonies were identified to the species level using matrix assisted laser desorption/ionization time-of-flight mass spectrometry (Bruker Daltonik GmbH, Bremen, Germany). Confirmed bacterial isolates were stored at -80 °C in 25% glycerol supplemented Tryptone soya broth (TSB) (CM0129, Thermo Scientific™, Australia) [28].

### Antimicrobial susceptibility testing of clinical bacterial isolates

Antimicrobial susceptibility was studied using the Kirby-Bauer disc diffusion method to investigate the resistance pattern of clinical isolates as previously described [29]. Depending on the type of isolates 15 antibiotics from more than seven different classes were tested (Supplementary Table 1). The zone of inhibition was measured using a ruler, and the results were interpreted as either sensitive or resistant based on the 2025 EUCAST break point values (EUCAST, 2025) and published literature for not specified by either EUCAST or CLSI [30]. Details on interpretation of methicillin and mupirocin resistant *Staphylococcus* species has been described in Supplementary Method 1 and 2. Isolates resistant to at least three classes of antibiotics were considered as MDR [31]. *E. coli* ATCC 25,922, *S. aureus* ATCC 25,923 and *S. aureus* ATCC 43,300 were used as a quality control strain.

### Whole genome sequencing (WGS) and bioinformatic analysis of selected isolates

Genomic DNA was extracted from a representative of the most predominant species, *S. aureus*, and most MDR species, *S. capitis*, for WGS. DNA extracts were stored at -20 °C before sent to Australian Genome Research Facility (AGRF) in Melbourne, Australia for WGS sequencing. Detailed information on library preparation and sequencing performed by AGRF, as well as the subsequent analysis of raw reads using the TORMES pipeline (v1.4) [32], is provided in Supplementary Method 3.

### Genomic DNA extraction for 16S rRNA sequencing

Genomic DNA was extracted from wound swab samples using the Invitrogen PureLink^Tm^ Microbiome DNA purification kit Catalogue number A29790 (Thermo Fisher Scientific Australia Pty Ltd, VIC Australia, ) following manufacturer’s instructions. DNA extracts were then stored at − 20 °C before being sent to Australian Genome Research Facility (AGRF) (Queensland, Australia) for 16S rRNA sequencing.

### Full-length 16S rRNA sequencing and bioinformatic analysis

High fidelity (HiFi) full-length 16S rRNA sequencing using Pacific biosciences (PacBio) technology was performed by AGRF, following in house protocols [33]. Bioinformatic data generated using QIIME2 pipeline [34] by AGRF were utilised to perform downstream microbial community analysis using the Phyloseq platform in RStudio (R, version 4.2.0) [35] (Supplementary Method 4).

### Genomic data availability

Whole genome and full-length 16S rRNA sequencing data have been deposited in the NCBI database under Bio Project number PRJNA1305620 and PRJNA1305565, respectively. They can be accessed using the following BioSample accessions: SAMN50612057, SAMN50612058, SAMN50611267, SAMN50611268, SAMN50611269, SAMN50611270, SAMN50611271, SAMN50611272, SAMN50611273, SAMN50611274, SAMN50611275, and SAMN50611276.

## Results

### Australian EB patient characteristics and wound microbiology

**Fig. 1:**
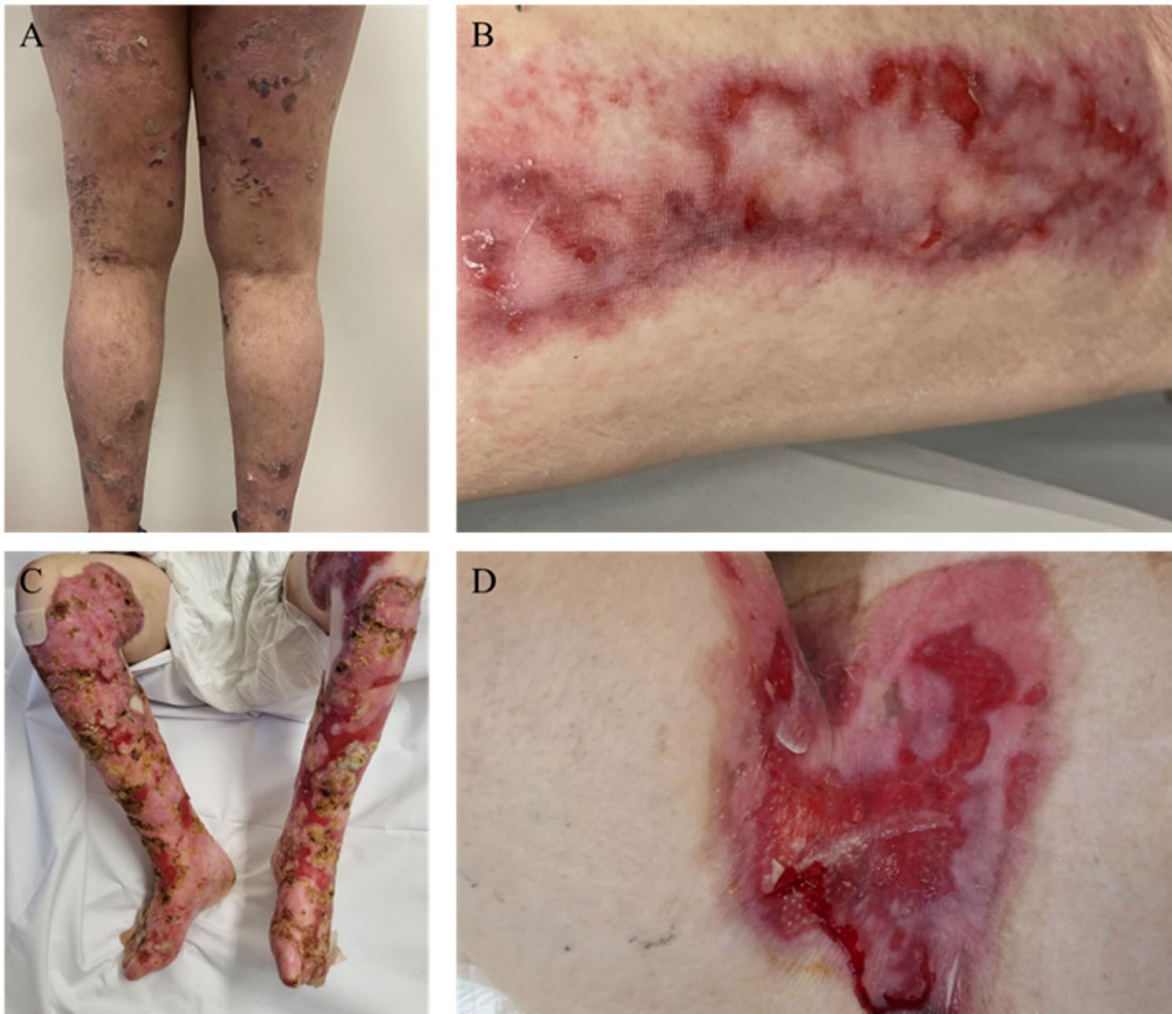
Representative photograph of wounds from which swab samples were collected. (**A**) EBS left foot and leg (**B**) dominant DEB right thigh, (**C**) JEB bilateral lower legs, (**D**) recessive DEB left groin

Ten patients (6 males and 4 females) with EB wounds were enrolled in this study. Almost all patients were adults, with a mean age of 37.6 years (range: 17–66 years). Among the 10 EB patients, 40% (4/10) had JEB, 30% (3/10) had EBS and 30% (3/10) DEB subtype with various severity of clinical presentation (Fig. 1).

Genetic analysis was performed for 80% (8/10) EB patients, while the remaining two had histological evidence supporting their EB-subtype diagnosis. Subtype-specific mutations were confirmed as EBS patients harboured *KRT14* mutations or histological keratin abnormalities, JEB patients had pathogenic variants in *LAMB3*,* LAMC2*,* LAMA3*, and *COL17A1*, and *DDEB/RDEB* patients carried *COL7A1* mutations, including compound heterozygous variants. Notably, siblings EB03 and EB04 shared identical mutations, consistent with their similar clinical presentations, although wound location and extent varied (Table 1).

As expected, EBS patients generally had lower wound body surface area (WBSA) involvement (1–22%), although one severe EBS case demonstrated more extensive skin involvement (22%), consistent with greater disease severity. JEB patient exhibited moderate WBSA values (7-25.5%), reflecting the more extensive and persistent nature of wounds. Dystrophic EB (DDEB and RDEB) generally exhibited lower WBSA values (4–8%), although one DDEB patient had SCC (Table 1).

Of the 15 wound swabs collected from the 10 EB patients at different locations and times, 13 swabs were colonised/infected by one or more bacterial species (*n* = 27 from 8 genera). The genus *Staphylococcus* was the most prominently identified microorganism, accounting for 48.1% (13/27). Within this genus, *S. aureus* was the most prevalent species, accounting for 38.5% identified isolates (5/13). Gram-negative bacteria accounted for one-quarter of the isolates 25.9% (7/27), recovered from 3 different patients (Table 1). These findings are consistent with the result obtained from local clinical microbiology laboratory, supporting the reliability of the culture-based identification (Supplementary Table 2). However, it is worth noting that the local microbiology laboratory did not further characterise mixed culture result and skin commensals to the species level nor determined the antimicrobial resistance profiles.

Overall, WBSA varied widely across EB subtypes and individuals, ranging from 1% to 25.5%. Higher WBSA values were more frequently observed in JEB and severe EBS cases, which could impact wound microbial burden. Patients with JEB, RDEB and severe EBS harboured both Gram-positive and Gram-negative organisms, whereas EBS patients with lower WBSA predominantly grow Staphylococcus species.

**Table 1:** Demographic and clinical characteristics of 10 EB patients and microorganisms isolated from wounded skins

ID	Age	Sex	EB subtype	Genetic mutation (s)	SCC	Wound site	WBSA (%)	16S sequence	Bacterial isolates using culture
EB01A	35	M	EBS (sever)	Keratin 14 in codon 119 (M119T)^a^	No	Left foot	22%	Yes	*Proteus mirabilis*
									*Brevundimonas diminuta*
									*Alcaligenes faecalis*
EB01B						Left leg		Yes	*Streptococcus pyogenes*
EB02A	47	M	DDEB	Collagen 7 A mutation of exon 73 (G2043R) hotspot	No	Right leg	8		*Dermacoccus nishinomiyaensis*
EB02B						Left leg		Yes	No growth
EB03A	24	M	JEB	1 A intermediate, homozygous for a splice variant in the intron 9 of LAMB3, immunofluorence mapping: reduced LAMB3, LAMC2 and LAMA3 which suggests inframe deletion	No	Left leg	13	Yes	*Staphylococcus aureus*
EB03B			(Non Herlitz)			Left thigh		Yes	*Staphylococcus aureus*
EB04	17	F	JEB	Siblings with EB03: exact same genetic mutations	No	Right breast	14	Yes	No growth
EB05	66	M	DDEB	Heterozygous for the variant COL7A1:c6191G > A (Gly2064Glu)	Yes	Right thigh	4	Yes	*Staphylococcus capitis*
EB06A	29	M	JEB	LAMC2 mutation- deletions affecting exon 9 and intron 9. COL17A1 exon 52 (homozygous A/A, experimental finding)	No	Right leg	25.5	Yes	*Staphylococcus aureus*
			(Non Herlitz)						*Streptococcus dysgalactiae*
EB06B						Left leg		Yes	*Corynebacterium striatum*
									*Staphylococcus lugdunensis*
EB06C						Left elbow		No	*Staphylococcus aureus*
									*Corynebacterium striatum*
EB07	53	F	EBS	No genomic testing. Histology report: ‘most likely keratin 5/6 and 14 abnormality’.	No	Sole	1	No	*Staphylococcus epidermidis*
									*Staphylococcus warneri*
EB08*	8	M	JEB	Genetic mutation laminin alpha3a genes, one of the classical isoform and other relatively newly discovered isoform^b^	No	Buttock	7	No	*Proteus mirabilis*
									*Pseudomonas aeruginosa*
EB09	57	F	EBS	No genomic testing. Histology report: EBS	No	Right leg	4	No	*Staphylococcus lugdunensis*
									*Staphylococcus haemolyticus*
EB10	24	F	RDEB	2 mutations identified in the type VII Collagen gene. The maternal mutation designated 3478delG is a single base deletion in exon 26 and the paternal mutation G2775S results in a transition from a G to A transition in exon 112	No	Left groin	7	No	*Streptococcus dysgalactiae*
									*Corynebacterium striatum*
									*Pseudomonas aeruginosa*
									*Alcaligenes faecalis*
									*Staphylococcus simulans*
									*Staphylococcus aureus*
									*Staphylococcus haemolyticus*

Note: * EB08 patient had history of hospitalised and antibiotic use, EB06C swab sample was collected one year after the first swab collection (EB06A&B). EB: epidermolysis bullosa, WBSA (%): wound body surface area percentage was estimated using the patient’s palm as a practical guide for approximating wound area, SCC: squamous cell carcinoma, EBS: EB simplex, JEB: junctional EB, DDEB: dominant dystrophic EB, RDEB: recessive dystrophic EB

a, Cummins RE, Klingberg S, Wesley J, Rogers M, Zhao Y, Murrell DF. Keratin 14 point mutations at codon 119 of helix 1 A resulting in different epidermolysis bullosa simplex phenotypes. JID. 2001;117 [5]:1103−7

b, Cohn HI, Murrell DF. Laryngo−onycho−cutaneous syndrome. Dermatologic clinics. 2010;28 [1]:89–92

### PacBio full-length 16S rRNA sequencing reveals dominance of *Staphylococcus and Streptococcus* in wounds of Australian patients with EB

**Fig. 2:**
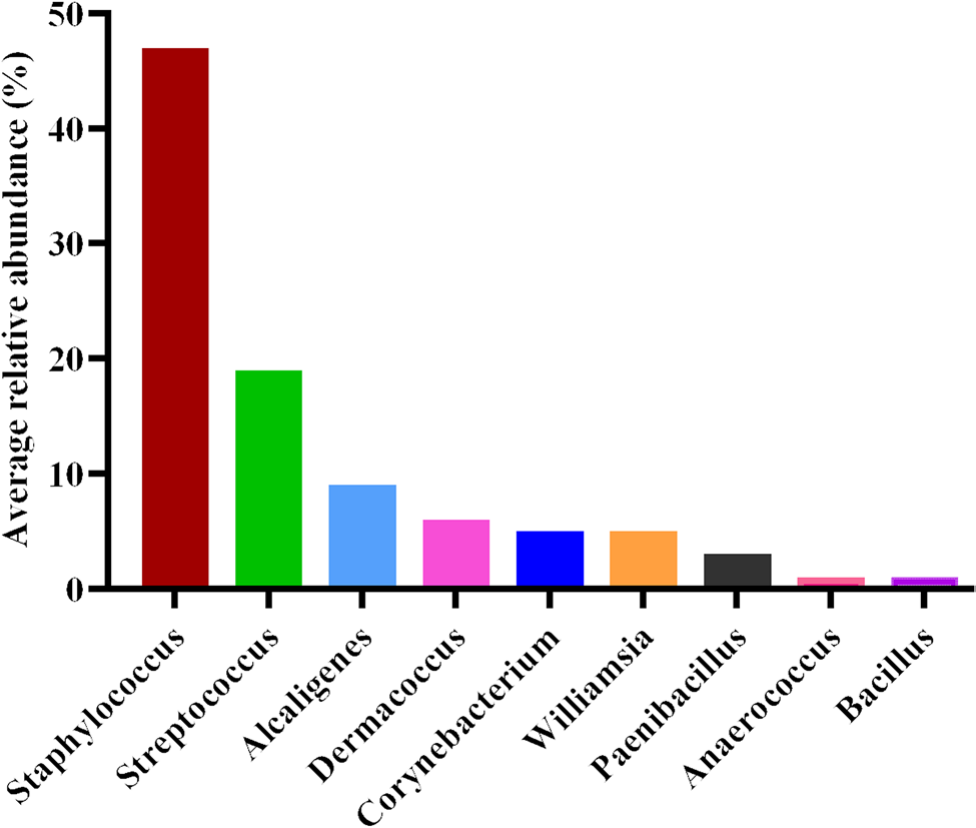
Relative abundance of dominant bacterial genera in wounds of Australian patients with EB (PacBio16S). Only genera with ≥ 1% relative abundance in at least two samples are displayed on bar plots

**Fig. 3:**
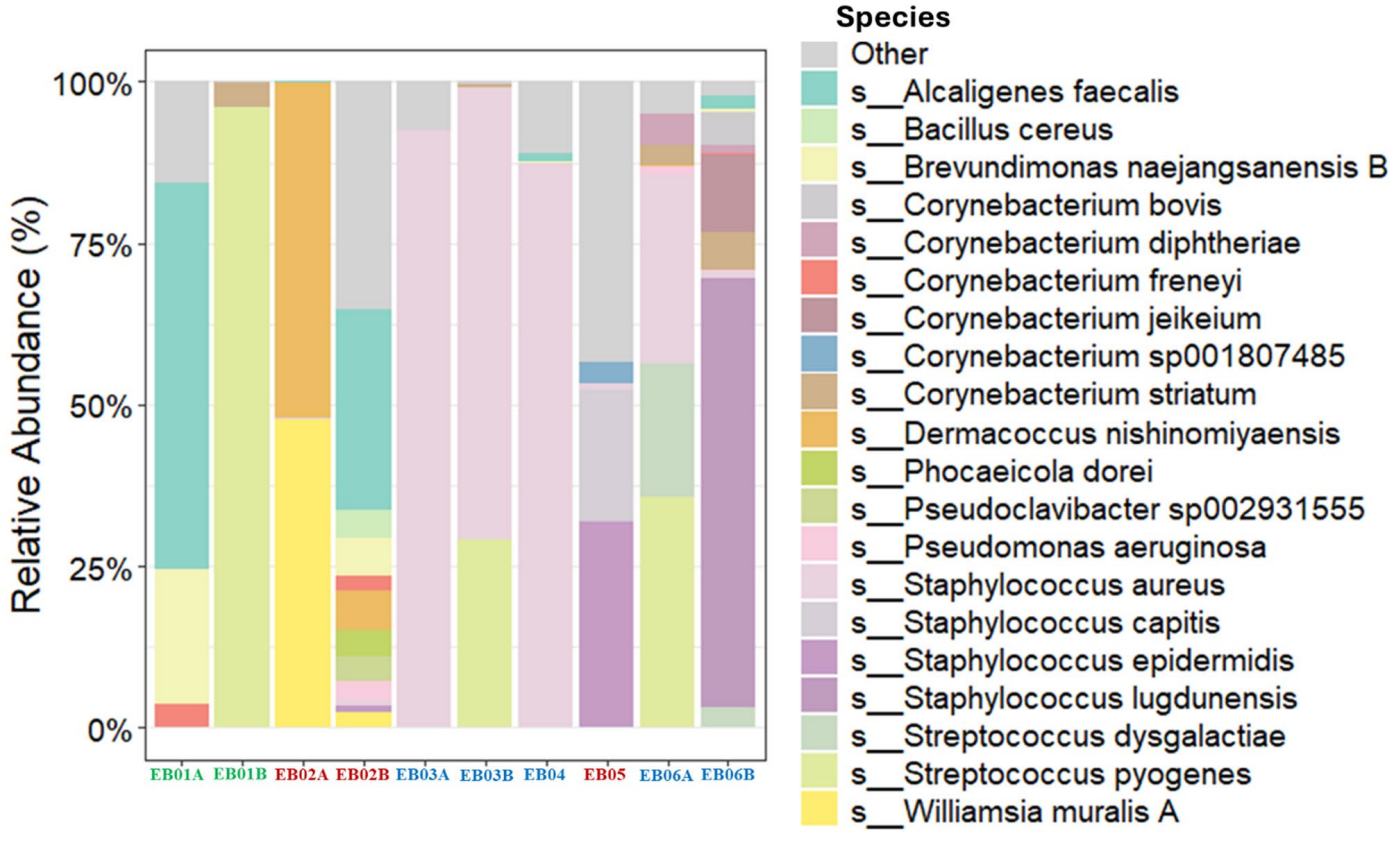
Relative abundance of the top 20 bacterial species identified in 10 EB wound swabs. EB subtypes were colour coded as green for EBS, blue for JEB and red for DEB

Full length 16S rRNA gene sequencing was performed on the first ten wound swabs listed in Table 1 from six Australian patients with EB. Taxonomic analysis identified nine predominant bacterial genera with relative abundance of ≥ 1% in at least two samples. Among these, *Staphylococcus and Streptococcus* were the most dominant, comprising 47% and 19% of the total bacterial community, respectively (Fig. 2). The top five most abundant genera identified via 16S rRNA gene sequencing were also detected by routine culture methods in corresponding swab samples, apart from sample EB02B and EB04 (Table 1; Fig. 3). In contrast, the remaining four most abundant genera *Williamsia*,* Paenibacillus*,* Anaerococcus* and *Bacillus* were detected exclusively by 16S rRNA gene sequencing.

All sequenced wound samples contained members of the *Staphylococcus* genus, with relative abundances ranging from 3% to 98%, and particularly exceeding 60% in JEB patients (EB03A&B, EB04 and EB06A&B). Within this genus, *S. aureus* was the most prevalent species, comprising over 50% of the total bacterial population in 5 out of the 10 samples (Fig. 3). Notably, in the EB04 wound sample, 16S rRNA sequencing revealed a dominant presence of *S. aureus* (> 90% relative abundance), yet no bacterial growth was detected using standard culture techniques (Fig. 3). Overall, the discrepancies observed at both the genus and species taxonomic levels highlight the limitations of culture-based methods in capturing the full diversity of the wound microbiome.

To further investigate microbial differences among EB subtypes (EBS, JEB and DEB), alpha and beta diversity analyses were performed (Supplementary Table 1A-F). No significant differences were observed in bacterial richness or diversity within samples based on Observed species or Simpson and Shannon indices (*p* > 0.05) (Supplementary Fig. 1A-C). To assess overall differences in bacterial community composition between EB subtypes, a permutational multivariate analysis of variance (PERMANOVA) using Bray-Curtis dissimilarity was conducted. The result suggested a modest difference in microbial composition across EB subtypes (DEB, EBS and JEB), although this did not reach statistical significance (F = 1.60, R^2^ = 0.313, *P* = 0.052) (Supplementary Fig. 1F). The observed R^2^ value suggests that 31.3% of the variation in microbial composition could be attributed to EB subtype, indicating a potential trend toward subtype-specific microbial profiles that warrants further investigation with a larger sample size.

### Prevalence of antimicrobial resistance in EB wound isolates from Australia

**Fig. 4:**
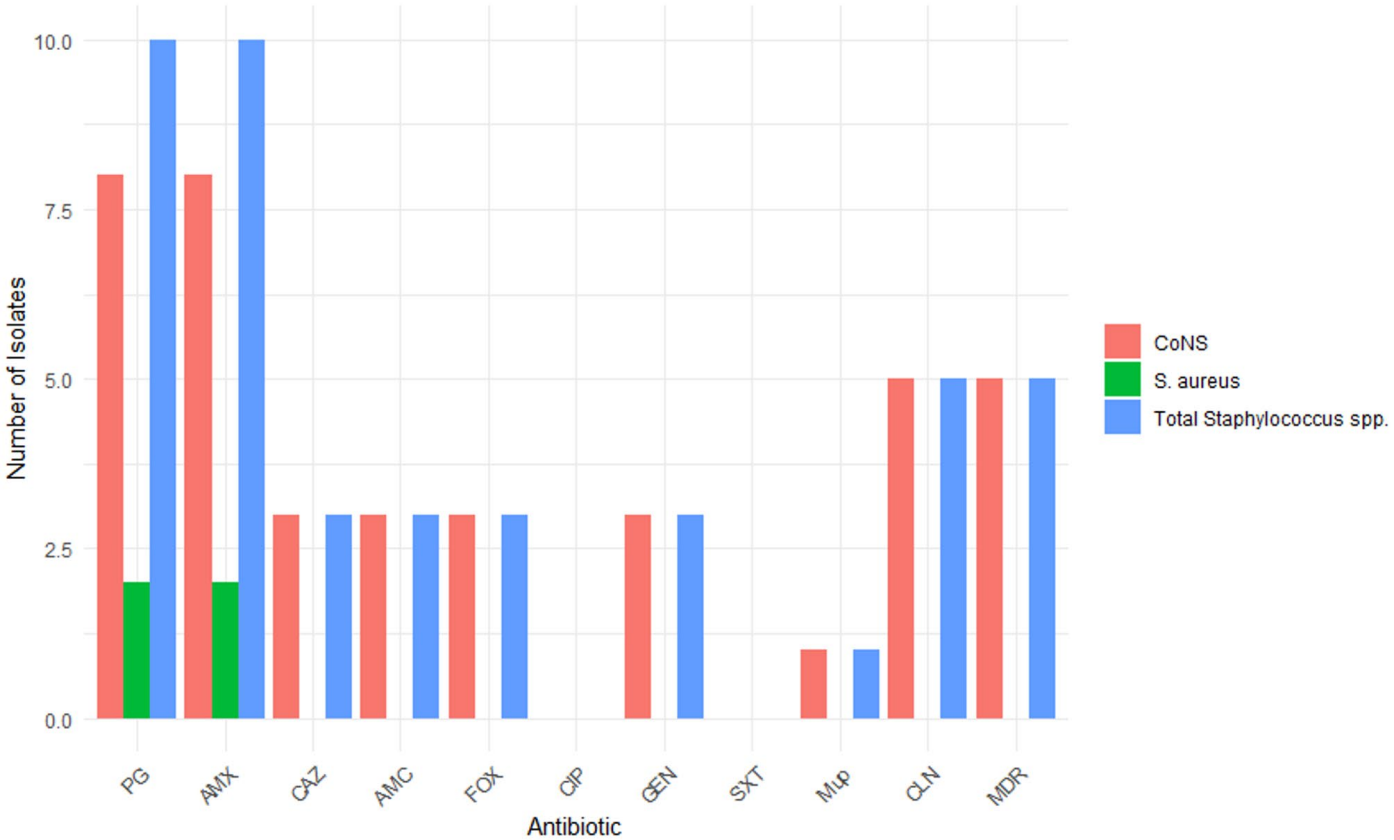
Antimicrobial resistance patterns of *Staphylococcus* species isolated from EB wounds. PG: Penicillin, AMX: amoxicillin, AMC: amoxicillin clavulanic acid, CAZ: cefazoline, FOX: cefoxitin, CIP: ciprofloxacin, GEN: gentamicin, and SXT: trimethoprim-sulfamethoxazole, Mup: mupirocin, CLN: clindamycin, MDR: multidrug resistance

To assess the antimicrobial resistance profile of all 27 clinical isolates a disk diffusion assay against various classes of antibiotics, depending on the type of isolate, was used. Overall, 81.5% (22/25) of isolates were resistant to at least one antimicrobial agent, and 22.2% (6/27) were classified as multidrug resistance (MDR). In contrast, 18.5% (5/27) of isolates were sensitive to all tested antimicrobial agents (Supplementary Table 3). *Staphylococcus* species were the most frequently isolated bacteria, comprising 48.1% (13/27) of all isolates. Among these, 76.9% (10/13) were resistant to penicillin and amoxicillin, followed by 38.5% (5/13) demonstrating resistance to clindamycin (Fig. 4). Resistance to cefoxitin, a surrogate marker for methicillin resistance, was observed in 3 out of 13 *Staphylococcal* isolates (23.1%), corresponding to 3 out of 10 patients (30%) in the cohort (Fig. 4). These cefoxitin-resistant isolates, identified as *S. capitis*,* S. epidermidis* and *S. haemolyticus*, were classified as Methicillin-resistant coagulase-negative Staphylococci (MR-CoNS). Of particular concern, one *S. capitis* isolate exhibited high-level resistance to mupirocin, a commonly used topical antibiotic in EB wound care. Although there were no established EUCAST or CLSI interpretive criteria, nor published literature defining susceptibility break points for the antibiotics tested against *Dermacoccus nishinomiyaensis* at the time of this study, all tested antimicrobial agents demonstrated considerable zone of inhibition.

### Whole genome sequencing analysis confirmed the phenotypic resistance profiles of selected resistant strains

**Fig. 5:**
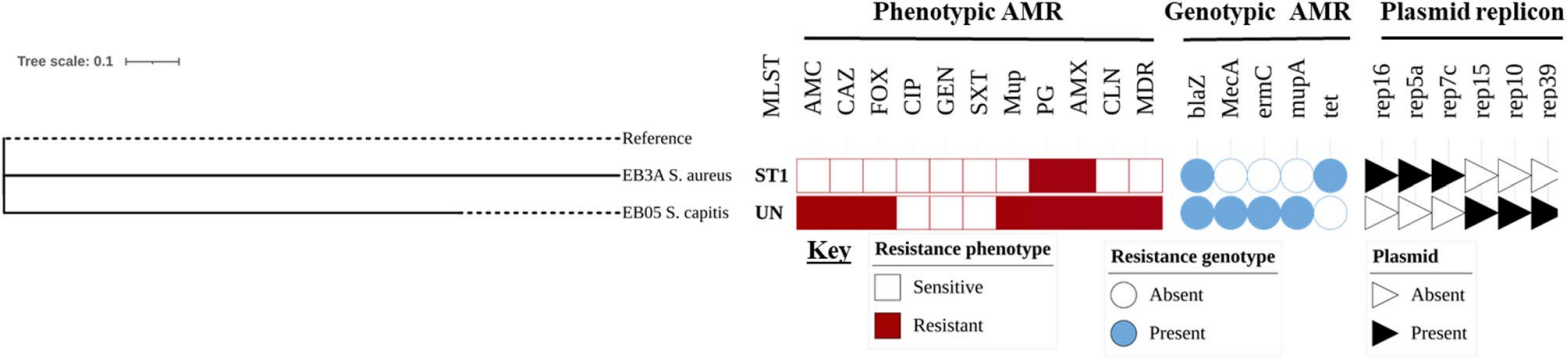
WGS-based MLST, resistance determinants, and plasmid replicons in two AMR Staphylococcus species from EB patients. A complete genome of Staphylococcus capitis subsp. capitis (accession number: GCA_001028645.1) was used as the reference genome. AMC: amoxicillin clavulanic acid, CAZ: cefazoline, FOX: cefoxitin, CIP: ciprofloxacin, GEN: gentamicin, and SXT: trimethoprim-sulfamethoxazole, Mup: mupirocin, PG: Penicillin, AMX: amoxicillin, CLN: clindamycin, MDR: multidrug resistance, MLST: multi-locus sequence type, UN: unknown

WGS was performed on selected AMR strains shown in Fig. 5 to elucidate their genomic resistance profiles and presence plasmid replicons. The sequence analysis confirmed the presence of resistance genes corresponding to the observed phenotypic resistance patterns against various antimicrobial agents **(**Fig. 5**)** and identified genes linked to biofilm formation and multiple virulence factors know to contribute to pathogenicity and host immune evasion (Supplementary Table 4). Both strains carried the *bla*Z gene, which encodes the penicillinase responsible for conferring resistance to penicillin-based antibiotics. In addition to tested antibiotics, the *S. aureus* EB03A isolate, belonging to sequence type 1 (ST1), also harboured the *tet* gene, conferring resistance to tetracycline. Notably, the commensal organism *S. capitis* EB05, with an undetermined ST, carried multiple genes conferring resistance to several of tested antimicrobial agents. Along with the *bla*Z gene, this isolate possessed the *mec*A gene, responsible for conferring resistance to several beta-lactam drugs including combinations with beta-lactamase inhibitor e.g., amoxicillin-clavulanic acid. This isolate also carried other genes, including *erm*C and *mup*A, conferring resistance to macrolides and mupirocin (a common topical agent), respectively. Furthermore, the coexistence of multiple plasmid replicons, antimicrobial resistance genes and virulence determinants in both isolates underscores the clinical challenges posed by these strains. The presence of plasmid replicons is particularly concerning, as they facilitate horizontal transfer of resistance genes, potentially exacerbating the dissemination and persistence of AMR in the clinical settings.

## Discussion

Although, infection management in EB is necessarily highly individualised, a better understanding of the broader microbial landscape in which patients live may support individualised care by identifying clinically relevant organisms in different settings and informing preparedness for emerging pathogens or antimicrobial resistance patterns. This study analyses the wound microbiome and antimicrobial resistance (AMR) in patients with EB in Australia using standard culture-based methods and full-length 16S rRNA gene sequencing.

In this study, microbiological profiling of 15 wound swabs from 10 EB patients revealed a predominance of *Staphylococcus* species (48.1%). This finding was supported by full-length 16S rRNA sequencing of the first 10 swab samples from 6 patients, which showed a relative abundance of *Staphylococcus* (47%) followed by *Streptococcus* (19%). This finding aligns with previous reports from both EB [14, 21, 22, 24] and non-EB chronic wound studies [36], where these genera consistently associated with wound colonisation and infection due to the opportunistic pathogenicity and biofilm-forming capabilities. The predominance of *Staphylococcus*, particularly *S. aureus*, is clinically relevant in the context of EB wound infections given its known association with delayed healing, persistent inflammation and antibiotic resistance [24, 37]. Recent studies have also highlighted the complexity of EB-associated pathogenicity, particularly in RDEB, where patients exhibit heightened immune activation alongside immune cell exhaustion [38]. Additionally, skin-adapted *S. aureus* from severe RDEB wounds have been shown to influence immune responses and clinical severity, with affected patients demonstrating an IL-17-skewed immune response explaining the variable virulence potential of bacterial clinical isolates [39].

Overall, the most abundant aerobic and facultative anaerobic bacteria identified by 16S rRNA sequencing were generally consistent with those recovered by standard culture methods. An exception was observed in sample EB04, which showed no bacterial growth on culture, despite 16S rRNA sequencing revealed over 90% sequence identity to *S. aureus.* This discrepancy highlights the greater sensitivity of molecular techniques. The absence of *S. aureus* growth in this case may be attributable to sequential wound swabbing, which could have partially depleted bacterial biomass because of prior swabbing for local microbiology culture, illustrating a limitation of routine culture-based methods. Together, these observations support the complementary role of sequencing-based approaches in more comprehensive characterisation of the EB wound microbiota.

Although *P. aeruginosa* is commonly reported in chronic wounds, its relative abundance in this study did not reach the predefined threshold of ≥ 1% in at least two samples (Fig. 2). This may reflect the small sample size and wound heterogeneity, supported by our culture result in which *P. aeruginosa* was identified in one JEB and one RDEB patients whose wound swabs were not subjected to 16S rRNA sequencing. Nevertheless, *P. aeruginosa* was among the top 20 species detected across EB patients, indicating its presence despite low relative abundance (Fig. 3).

Antimicrobial resistance is recognised a contributing factor to delayed wound healing, persistent infection, and treatment failure in chronic wounds, including those associated with EB [17]. In this study, a high proportion of AMR positive isolates (81.5%), including MDR organisms (22.2%), was observed, indicating ongoing therapeutic challenges in EB wound management. Methicillin resistance was identified in 23% of *Staphylococcus* isolates, a prevalence comparable to Australian national data estimates (15–19%) [40], but lower than rates reported in some international EB cohorts, like the United States, where MRSA prevalence has been reported to reach 47% [15]. This discrepancy may reflect the longstanding caution among Australian doctors regarding mupirocin use in EB patients, with its recommendation significantly reduced over the past two to three decades in response to high resistance levels reported internationally. Several studies have demonstrated that frequent and prolonged use of mupirocin can lead to the selection of multidrug-resistant MRSA strains, potentially due to co-selection pressure involving resistance genes carried on the same mobile genetic elements [41, 42].

One MDR *S. capitis* isolate (EB05), recovered from a 64-year-old patient with DDEB and SCC, exhibited methicillin resistance and high-level mupirocin resistance. This observation is consistent with reports suggesting that the development of MDR phenotypes may increase with patient age and cumulative antimicrobial exposure [43]. High-level mupirocin resistance remains clinically relevant, given the continued use of this topical agent in wound care. These findings align with reports by Bathoorn et al., which linked increased short-term mupirocin use to the emergence of high-level resistance in CoNS [44]. Moreover, previous studies have also demonstrated associations between methicillin resistance and biofilm formation in wound isolates including *S. capitis* [45, 46]. WGS analysis of the *S. capitis* EB05 isolate confirmed the presence of multiple AMR genes, including *blaZ*,* mecA*,* ermC*,* mupA*, consistent with the observed phenotypic resistance. This isolate also harboured the *ica* operon, associated with biofilm formation [47] and plasmid replicons known to carry resistance antimicrobial resistance gene and multiple virulence factors [48]. These findings suggest that EB wounds may serve as reservoirs for resistance genes, particularly in chronic or recurrently infected wounds.

In contrast to reports from some international cohorts, none of the *S. aureus* isolates in this Australian EB cohort were identified as MRSA [15, 17, 19]. This may reflect the small sample size; however, two *S. aureus* isolates from different anatomic sites were resistant to other beta-lactam antibiotics including penicillin and amoxicillin. One of these isolates, subjected for WGS analysis carried resistance genes conferring phenotypic resistance, quorum sensing regulatory gene and multiple virulence factors, suggesting its potential for persistence and pathogenicity despite methicillin susceptibility (Supplementary Table 4). While the small sample size limits identification of EB subtype-specific trends, all subtypes demonstrated reduced microbial diversity, with clinical isolates from JEB patients exhibiting the highest abundance of *Staphylococcus* species, in particular *S. aureus.* Among MRCoNS, two of three isolates were identified in EBS patient samples and one of three in DEB patient sample. Together, these findings suggest that stronger engagement within EB multidisciplinary teams regarding appropriate use of oral and topical antibiotics and dressings from birth onwards is required to decrease high levels of antimicrobial resistance in EB patients. Additionally, the potential impact of off-label treatments and biologic therapies aimed at reducing inflammation should be considered in relation to their effects on wound microbiome in EB patients.

While this study offers important insights, it has some limitations. First, the sample size was small (*n* = 10), due to the rarity of EB, which limits the generalisability of the findings and precludes robust statistical analysis across different EB subtypes. Recruiting a larger and more diverse cohort remains challenging, particularly for rare EB variants. Nevertheless, swab samples were collected irrespective of wound healing stage, enabling isolation of a diverse bacterial population for antimicrobial susceptibility testing. Second, although this study employed full-length 16S sequencing of wound swabs and WGS of selected resistant isolates, whole genome metagenomic sequencing of wound swab samples could offer deeper insights into functional microbial genes, and AMR composition in future studies. Finally, this work could be extended to paediatric EB population to assess its impact on clinical infection rates and the development of antimicrobial resistance.

## Conclusion

This study describes the wound microbiota and antimicrobial resistance patterns in Australian EB patients using complementary culture-based and molecular approaches. Staphylococcus species were predominant, and antimicrobial resistance including MDR was commonly observed. The addition of 16S rRNA sequencing improved detection of clinically relevant organisms that were not consistently recovered by routine culture. Interpretation of the findings is limited by the small sample size, cross-sectional design, and sequencing of subset of isolates. Nevertheless, these findings support the value of integrating molecular methods with standard microbiology and highlights the need for future studies incorporating larger, longitudinal cohort and paediatric populations to better define the relationship between wound microbiota, antimicrobial exposure and clinical outcomes over time.

## Supplementary Information

Below is the link to the electronic supplementary material.

Supplementary Material 1

## Data Availability

All data generated or analysed during this study are included in this published article and its supplementary information files.
