# Supplementary material for: Characterisation of the wound microbiome and antimicrobial resistance profiles in clinical isolates from epidermolysis bullosa patients
Source: Orphanet J Rare Dis. 2026 Mar 7;21:151. doi: 10.1186/s13023-026-04295-5 (PMC13081397; doi:10.1186/s13023-026-04295-5)
Supplement: Supplementary file 1 — Supplementary Material 1 [file 13023_2026_4295_MOESM1_ESM.docx]

**Characterisation of the wound microbiome and antimicrobial resistance profiles in clinical isolates from Epidermolysis Bullosa patients**

*Anteneh Amsalu^1^, Hanif Haidari ^2^, Bianca Mirco^3^,* *Victoria Rudolph-Stringer^3^, Anna Antipov^1^, Dédée F Murrell^3,4^ and Zlatko Kopecki^1^**

^1^ Future Industries Institute, University of South Australia, Mawson Lakes, SA 5095, Australia

^2^ College of Medicine and Public Health Flinders University, Bedford Park SA 5042, Australia

^3^ Department of Dermatology, St. George Hospital, Sydney, Australia

^4^ Faculty of Medicine, University of New South Wales, Sydney, Australia.

*Corresponding author

Dr Zlatko Kopecki: [zlatko.kopecki@unisa.edu.au](mailto:zlatko.kopecki@unisa.edu.au)

**Materials and Methods**

**Supplementary Table 1.** Antibiotics used for testing susceptibility of clinical isolates.

| **Antibiotic class** | **Type of antibiotic used** | **Concentration tested** |
| --- | --- | --- |
| **Β-lactams** | Penicillin | 1IU |
|  | Amoxicillin | 3µg |
|  | Cefazoline | 30µg |
|  | Cefazoline | 30µg |
|  | Cefepime | 30µg |
|  | Imipenem | 10µg |
|  | Amoxicillin -clavulanic acid | 20/10µg |
|  | Piperacillin-tazobactam | 110µg |
| **Macrolides** | Clindamycin | 2μg |
| **Fluoroquinolones** | Ciprofloxacin | 5µg |
| **Aminoglycosides** | Gentamycin | 10 µg |
|  | Tobramycin | 10 µg |
| **Glycopeptides** | Vancomycin | 30µg |
| **Miscellaneous agents** | Trimethoprim - sulfamethoxazole | 1.25/23.75µg |
|  | Mupirocin | 200µg |

1. **Phenotypic detection of MRSA and MRCoNS**

Susceptibility of Staphylococcus species to cefoxitin was used to detect the methicillin-resistance phenotype. Inhibition zone of < 22mm and 27mm in diameter for *S. aureus* and *Coagulase*-negative *Staphylococci* (CoNS) were interpreted as Methicillin-resistant *S. aureus* (MRSA) and Methicillin-resistant-*CoNS* (MR-*CoNS*), respectively according to the EUCAST breakpoint, 2025. *S. aureus* ATCC 25923 and *S. aureus* ATCC 43300 were used as a negative and positive control, respectively for cefoxitin inhibition assay.

1. **Phenotypic detection of high-level mupirocin resistance among *Staphylococcus* species**

All isolated *Staphylococcus* species were tested for mupirocin (200μg) using a disk diffusion assay. Isolates that exhibited no zone of inhibition were classified as having high-level mupirocin resistance (MUP^H^) (1), while those displaying any measurable zone of inhibition around the disc were considered absence of MUP^H^ resistance.

1. **Whole genome sequencing (WGS) and bioinformatic analysis of selected isolates**

Genomic DNA was extracted from a representative sample of the most predominant species, *S. aureus*, and most MDR species, *S. capitis*, for WGS. A pure single colony of selected bacterial isolate was incubated in 10mL of MHB at 37°C. After overnight growth, bacterial cells were harvested by centrifugation at 5000g for 10min. The supernatants were discarded, and the pallets were used for genomic DNA extraction using Invitrogen PureLink^Tm^ Microbiome DNA purification kit (Thermo Fisher Scientific) following manufacturer’s instructions. DNA quantity and purity was evaluated spectrophotometrically using the Cytation5 imaging reader (BioTek Instruments, Winooski, Vermont, USA) and the Take3 Micro volume plate. DNA extracts were stored at -20°C before sent to Australian Genome Research Facility (AGRF) in Melbourne, Australia for WGS sequencing.

Prior to library preparation, DNA quality was verified using an E-Gel High Range Ladder. Libraries were prepared using Illumina DNA prep kit and sequenced on an Illumina NovaSeq platform, (Illumina Inc., USA), generating 150-base pair paired-end reads with Real Time Analysis (RTA) software. Read quality was assessed using FastQC to evaluate base quality scores, GC content, sequence duplication levels and other standard quality metrics (2). Reads were processed using the TORMES pipeline (v1.4) (3), an automated workflow for bacterial genome analysis. Within TORMES, reads underwent quality filtering with Prinseq (4) followed by *de novo* genome assembly using SPAdes (5) and genome annotation with Prokka (6). Multi-locus sequence typing (MLST) was performed using the MLST tool <https://github.com/tseemann/mlst> and antimicrobial resistance genes were identified using ABRicate (T. Seemann, https://github.com/tseemann/abricate) against the ResFinder (7), CARD (8) and ARG-ANNOT (9), databases. Virulence factors were detected using VFDB (10) and plasmid replicons were identified using PlasmidFinder (11) from the centre for genomic epidemiology online database. For phylogenetic analysis,  a core genome single nucleotide polymorphisms (SNPs) tree was constructed using CSI phylogeny 1.4 (https://cge.cbs.dtu.dk/services/CSIPhylogeny) (12), with the complete genome of *Staphylococcus capitis subsp. capitis* (accession number: GCA_001028645.1) used as the reference. The phylogenetic tree was visualised and annotated using Interactive Tree of Life (iTOL) (http://itol.embl.de) (13).

1. **Genomic DNA Extraction for 16S rRNA sequencing**

Genomic DNA was extracted from wound swab samples using the Invitrogen PureLink^Tm^ Microbiome DNA purification kit Catalogue number A29790 (Thermo Fisher Scientific Australia Pty Ltd, VIC Australia,) following manufacturer’s instructions. Negative controls were included throughout the DNA extraction procedure to assess potential contamination. DNA quantity and purity was evaluated spectrophotometrically using the Cytation5 imaging reader (BioTek Instruments, Winooski, Vermont, USA) and the Take3 Micro Volume plate. No measurable DNA was detected in the negative controls. DNA extracts were then stored at - 20°C before being sent to Australian Genome Research Facility (AGRF) (Queensland, Australia) for 16S rRNA sequencing.

**Full-length 16S rRNA library construction and sequencing**

High fidelity (HiFi) full-length 16S rRNA sequencing using Pacific biosciences (PacBio) technology was performed by AGRF, following in house protocols. Polymerase chain reaction (PCR) amplification of the entire 16S rRNA gene (V1-V9 regions) was performed using indexed primers F27 (5’GCATC/barcode/AGRGTTYGATYMTGGCTCAG3’) and R1492 (5’GCATC/barcode/RGYTACCTTGTTACGACTT3’). Kinnex full-length RNA kits were utilised in library preparation and SMARTbell paired-end-sequencing chemistry was used to obtain sequences to achieve high species-level taxonomic resolution (14).

**Bioinformatic analysis**

PacBio raw reads were quality filtered (15), trimmed with Cutadapt (16), followed by chimera removal and denoised using divisive Amplicon denoising Algorithm-2 (DADA2) to infer high-resolution amplicon sequence variants (ASVs) instead of operational taxonomic unit (OTU) (14).

Taxonomic assignment of ASVs was conducted using both VSERCH (which uses a single reference database) (17) and the naïve Bayes classifier which employed three databases including SILVA rRNA database (v138)(18), Genome Taxonomy Database (GTDB r207) (19), and the NCBI RefSeq 16S rRNA databases (20). These databases were supplemented by the ribosomal database project (RDP) to enhance the classification accuracy. Using QIIME2 pipeline (21), taxonomic assignment was performed at 97% sequence similarity level at the genus and species level, where possible.

The biological observation matrix (BIOM) file, termed “feature-table-biom” generated from QIIME2 was utilised to perform downstream microbial community analysis using the Phyloseq platform in RStudio (R, version 4.2.0) (22). To assess within-sample microbiota variation, alpha diversity was measured using Shannon, Simpson and observed feature diversity indices (23). Differences in alpha diversity among groups were evaluated using Kruskal-Wallis (KW) test. To assess microbial community composition between EB subtypes, beta diversity was analysed using Bray-Curtis dissimilarity, calculated from the rarefied ASV table. Community-level differences were tested using permutational multivariate analysis of variance (PERMANOVA) with 999 permutations, implemented via the adonis2() function in the vegan R package (24). Dissimilarities were visualised using a principal coordinates analysis (PCoA) based on the Bray-Curtis distance matrix. To further evaluate the effect of taxonomic abundance and phylogenetic relatedness, both unweighted and weighted UniFrac analyses were performed using QIIME2 (25). Almost all taxonomic profiling, diversity analyses, and data visualisation were performed using the Phyloseq, ggplot, and vegan packages within the RStudio platform environment (R, version 4.2.0).

**Results**


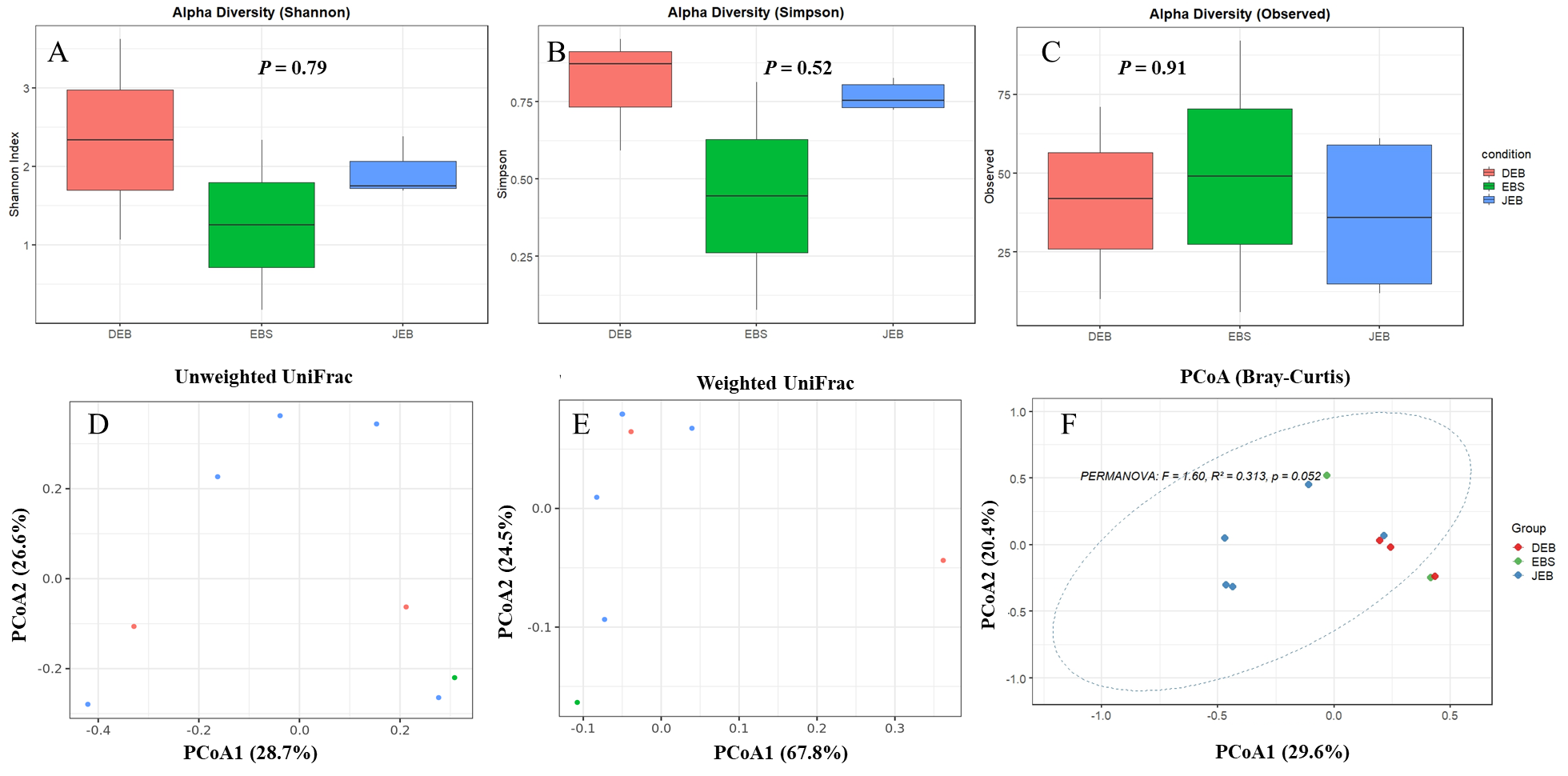


**Supplementary Figure 1.** Alpha and beta diversity analysis of the wound microbiota across EB subtypes. A) Shannon index, B) Simpson index, and C) the observed species richness are presented as boxplots for each subtype, with no statistically significant difference observed (P > 0.05, Kruskal-Wallis test). Beta diversity was assed using D) Unweighted UniFrac, E) weighted Unifrac, and F) Bray-Curtis dissimilarity metrics. The Unweighted and weighted UniFrac plots showed no clear phylogenetic clustering among the subtypes. However, principal coordinate analysis (PCoA) based on Bray-Curtis distances revealed modest separation of microbial communities by subtype, as indicated by the PERMANOVA test (P = 0.052).

**Supplementary Table 2.** Comparison of microbiology culture result between the two laboratories and list of antibiotics and anti-inflammatory agents used at the time of recruitment.

|  | | | | | **Wound culture result** | |
| --- | --- | --- | --- | --- | --- | --- |
| **Sr.No** | **Patient code** | **Body location** | **Drugs** | **Anti-inflammatory agents** | **Local Microbiology** | **UniSA Microbiology** |
| 1 | EB01-A | Left foot | No | Dapsone 25mg daily, ointment including triamcinolone, neomycin, gramicidin and nystatin PRN | *Arcanobacterium hemolyticum* and *Heavy growth of mixed coliform* | *Proteus mirabilis, Alcaligenes faecalis, Brevundimonas diminuta* |
|  | EB01-B | Left leg |  |  | *Streptococcus pyogenes* | *Streptococcus pyogenes* |
| 2 | EB02-A | Right leg | No | Dapsone 50mg daily, Colchicine 500mcg daily | *S. aureus* | *Dermacoccus nishinomiyaensis* |
|  | EB02-B | Left leg |  |  | Moderate growth of normal skin flora. | No growth |
| 3 | EB03-A | Left thigh | No      No | Colchicine 500mcg daily, Doxepin 10mg daily, ibuprofen oral med PRN | Moderate growth of *S. aureus* | *Staphylococcus aureus* |
|  | EB03-B | Right lower leg |  |  | Light growth of *S. aureus* | *Staphylococcus aureus* |
| 4 | EB04 | Right breast |  | Colchicine 500mcg daily, mometasone furoate cream PRN | Moderate growth of *S. aureus* | No growth |
| 5 | EB05 | Right thigh | No | No | Heavy growth of mixed skin flora. | *Staphylococcus capitis* |
| 6 | EB06-A | Right leg | No | Ivermectin 12mg twice weekly and topical prednisolone cream | Scanty growth of *S. aureus*, *Streptoccous dysgalactiae* | *Staphylococcus aureus, Streptococcus dysgalactiae* |
|  | EB06-B | Left leg |  |  | No growth | *Corynebacterium striatum,*  *Staphylococcus lugdunensis* |
|  | EB06-C | Left elbow | No | Ivermectin 12mg twice weekly, Colchicine 500mcg daily, and topical prednisolone cream x3 weekly | *S. aureus,* Moderate growth of normal skin flora | *Corynebacterium striatum,*  *Staphylococcus aureus* |
| 7 | EB07 | Sole | No | No | Light growth of skin flora | *Staphylococcus warneri, Staphylococcus epidermidis* |
| 8 | EB08 | Buttock | Doxycycline 100mg OD* | Dexamethasone, Thalidomide 100mg OD and diclofenac eyedrops daily, puffer including budesonide and formoterol daily | Heavy mixed growth including coliforms and Pseudomonas spp. | *Proteus mirabilis, P.aeruginosa* |
| 9 | EB09 | Right leg | Flucloxacillin ** PO |  | *S. aureus, S. lugdunensis* | *Staphylococcus lugdunensis, Staphylococus haemolyticus* |
| 10 | EB10 | Left Groin | No | Birch triterepene gel applied directly to EB wounds daily to 2^nd^ daily | *Staphylococcus aureus, Streptococcus dysgalactiae* and *Moderate mixed growth including Pseudomonas* | *Streptococcus dysgalactiae, Corynebacterium striatum,P.aeruginosa,Alcaligenes faecalis,Staphylococcus simulans,Staphylococcus aureus, Staphylococus haemolyticus* |

* EB08 was hospitalised for three days exactly two months prior to sample collection and was treated with intravenous (IV) ceftriaxone and azithromycin (IV) for left lower lobe pneumonia. Up on discharge, EB08 was prescribed a 7-day course of oral azithromycin and was receiving doxycycline 100mg once daily at the time of sample collection. ** EB09 received oral flucloxacillin for 5 days to treat non-healing blister on right ankle. Ointments are recommended to be applied only to the edges of wounds

**Supplementary Table 3.** Antimicrobial resistance profile of bacterial strains isolated from wounds of Australian EB patients.

| **ID** | **Site of wound** | **Bacterial Isolates** | **AMC** | **CAZ** | **FOX*** | **CIP** | **GEN** | **SXT** | **Mup** | **PG** | **AMX** | **CLN** | **MDR** |
| --- | --- | --- | --- | --- | --- | --- | --- | --- | --- | --- | --- | --- | --- |
| **EB01A** | Left foot | *Proteus mirabilis* | S | S | S | S | R | S | ND | ND | ND | ND | NMDR |
|  |  | *Brevundimonas diminuta* | S | S | S | R | S | S | ND | ND | S | ND | NMDR |
|  |  | *Alcaligenes faecalis* | S | R | S | S | S | S | ND | ND | ND | ND | NMDR |
| **EB01B** | Left leg | *Streptococcus pyogenes* | S | S | S | ND | ND | S | ND | S | S | S | S |
| **EB02A** | Right leg | *Dermacoccus nishinomiyaensis* | S S | | S | S | S | S | ND | S | S | ND | S |
| **EB02B** | Left leg | No growth |  |  |  |  |  |  |  |  |  |  |  |
| **EB03A** | Left leg | *Staphylococcus aureus* | S | S | S | S | S | S | S | R | R | S | NMDR |
| **EB03B** | Left thigh | *Staphylococcus aureus* | S | S | S | S | S | S | S | R | R | S | NMDR |
| **EB04** | Right breast | No growth |  |  |  |  |  |  |  |  |  |  |  |
| **EB05** | Right thigh | *Staphylococcus capitis* | R | R | R | S | S | S | R | R | R | R | MDR |
| **EB06A** | Right leg | *Staphylococcus aureus* | S | S | S | S | S | S | S | S | S | S | S |
|  |  | *Streptococcus dysgalactiae* | S | S | S | ND | ND | R | ND | S | S | S | NMDR |
| **EB06B** | Left leg | *Corynebacterium striatum* | S | S | S | R | R | R | ND | S | S | S | MDR |
|  |  | *Staphylococcus lugdunensis* | S | S | S | S | S | S | S | R | R | S | NMDR |
| **EB06C** | Left elbow | *Staphylococcus aureus* | S | S | S | S | S | S | S | S | S | S | S |
|  |  | *Corynebacterium striatum* | S | S | S | R | S | R | ND | S | S | S | NMDR |
| **EB07** | Sole | *Staphylococcus epidermidis* | R | R | R | S | S | S | S | R | R | R | MDR |
|  |  | *Staphylococcus warneri* | S | S | S | S | R | S | S | R | R | S | MDR |
| **EB08** | Buttock | *Proteus mirabilis* | R | R | R | S | S | S | ND | ND | ND | ND | NMDR |
|  |  | *Pseudomonas aeruginosa*** | R | R | S | S | ND | ND | ND | ND | R | ND | NMDR |
| **EB09** | Right leg | *Staphylococcus lugdunensis* | S | S | S | S | S | S | S | R | R | R | NMDR |
|  |  | *Staphylococus haemolyticus* | R | R | R | S | R | S | S | R | R | R | MDR |
| **EB10** | Left Groin | *Streptococcus dysgalactiae* | S | S | S | S | S | R | ND | S | S | S | NMDR |
|  |  | *Corynebacterium striatum* | S | S | S | S | S | S | ND | R | S | S | NMDR |
|  |  | *Pseudomonas aeruginosa* | ND | ND | ND | S | R | R | ND | ND | R | ND | NMDR |
|  |  | *Alcaligenes faecalis* | S | R | S | S | S | S | ND | ND | ND | ND | NMDR |
|  |  | *Staphylococcus simulans* | S | S | S | S | R | S | S | R | R | R | MDR |
|  |  | *Staphylococcus aureus* | S | S | S | S | S | S | S | S | S | S | S |
|  |  | *Staphylococus haemolyticus* | S | S | S | S | S | S | S | R | R | S | NMDR |

***Cefoxitin resistant isolates were further tested with Vancomycin, and all were found to be sensitive*. **P. aeruginosa* strains were tested for tobramycin, piperacillin- tazobactam, cefepime and meropenem according to the EUCAST guideline. Results showed both strains sensitive to the tested antibiotics. PG: Penicillin, AMX: amoxicillin, AMC: amoxicillin clavulanic acid, CAZ: cefazoline, FOX: cefoxitin, CIP: ciprofloxacin, GEN: gentamicin, and SXT: trimethoprim-sulfamethoxazole, Mup: mupirocin, CLN: clindamycin, FLU: Flucloxacillin/dicloxacillin, S: sensitive, R: resistant, NMDR: non-multidrug resistance, MDR: multidrug resistance.

**Supplementary Table 4.** Presence of virulence genes in Staphylococcus aureus EB03A and Staphylococcus capitis EB05 clinical isolates based on whole genome sequencing.

| **Function** | **Gene(s)** | **Product (s)** | ***Function*** | ***S. aureus EB03A*** | ***S. capitis EB05*** | ***Node***  ***(EB05)*** |
| --- | --- | --- | --- | --- | --- | --- |
| **Global regulator** | *agrADCB* | Accessory gene regulator (quorum sensing system) | Regulates toxin production and represses surface proteins; switches from colonization to invasion mode | Y | Y | EB05_02291  EB05_02294 |
|  | sarARVZ | Transcriptional activator | Regulate biofilm, toxin genes, and proteases; sarA activates biofilm-related genes like *icaADBC* | Y | Y | EB05_01142 |
|  | *sigB* | Sigma factor B | Stress response regulator; promotes biofilm, inhibits toxin production, enhances survival under harsh conditions | Y | Y | EB05_02111 |
|  | *saeRS* | Two component regulator system | Activates toxins like *hla* and *pvl* in response to host signals (e.g., neutrophils, low pH). | Y | Y | EB05_01053-  EB05_01054 |
|  | *arlRS* | Two component system | Involved in biofilm formation, autolysis, and resistance to stress. | Y | Y | EB05_00406-  EB05_00407 |
|  | *mgrA* | marR family transcriptional regulator | Controls autolysis, biofilm, antibiotic resistance; generally, represses biofilm formation |  |  | EB05_01073 |
| **Biofilm** | *icaRADBC* | Polysaccharide Intracellular adhesion (PIA) proteins | Encodes enzymes responsible for the synthesis of PIA, essential for biofilm matrix formation (*icaADBC*). Repressor of the *icaADBC* operon; controls PIA production (*icaR*) | Y | Y | EB05_01888 -EB05_01892 |
| **Capsular polysaccharide genes** | *capDACB (A-P)* | Capsular biosynthesis proteins | Encode enzymes involved in capsule synthesis (sugar metabolism, polymerization, and export) | Y | Y | EB05_01583-EB05_01587 |
| **Exoenzymes** | *aur* | zinc metalloproteinase aureolysin | Degrades host proteins, modulates complement and immune responses | Y | Y | EB05_00563 |
|  | *sspA* | Serine protease; V8 protease | Degrades immunoglobulins and structural proteins | Y | Y | EB05_00126 |
|  | *sspB* | Staphopain cysteine proteinase SspB | Degrades neutrophil components and ECM proteins. | Y | N |  |
|  | *sspC* | Staphostatin B (inhibitor) | Inhibits SspB to control proteolytic activity | Y | N |  |
|  | *sak* | Staphylokinase precursor | Activates plasminogen to plasmin → degrades fibrin clots, aids tissue invasion | Y | N |  |
|  | *Lip* | Triacylglycerol lipase precursor | Hydrolyzes triglycerides for nutrient acquisition | Y | Y | EB05_01893 |
|  | *hysA* | hyaluronate lyase precursor | Degrades hyaluronic acid in connective tissue; facilitates spread | Y | N |  |
|  | Coa | Staphylocoagulase precursor | Clots plasma → protects bacteria from phagocytosis. | Y | N |  |
|  | *vWbp* | Secreted von Willebrand factor-binding protein | Coagulase-like; promotes clotting and immune evasion | Y | N |  |
| **Proinflammatory peptides** | hly/hlb | Alpha-Hemolysin precursor | Forms pores in host cell membranes, leading to lysis of epithelial, endothelial, and immune cells; induces inflammation | Y | N |  |
|  | Hld | delta-hemolysin | Amphipathic peptide; contributes to cell lysis and inflammation; encoded within the RNAIII of the agr quorum sensing system | Y | N |  |
|  | Hlg | gamma-hemolysin | Form leukotoxins that target and lyse neutrophils, macrophages, and other immune cells; induce cytokine release | Y | N |  |
|  | *Sea* | Staphylococcal enterotoxin A precursor | Superantigens that non-specifically activate T cells → massive cytokine release → toxic shock syndrome, inflammation | Y | N |  |
|  | *Spa* | Immunoglobulin G binding protein A precursor | Binds Fc region of IgG; activates TNF-α release via Fcγ receptors and TLR2 in immune cells | Y | N |  |
| **Surface protein adhesion** | *fnbA, fnbB* | Fibronectin-binding protein A &B | Mediate attachment to fibronectin and host cells | Y | N |  |
|  | *clfA, clfB* | Clumping factors | Bind to fibrinogen and promote colonization. |  | N |  |
|  | *ebp* | Cell surface elastin binding protein | Promotes tissue colonization | Y | N |  |
|  | *sdrC* | Ser-Asp rich fibrinogen-binding bone sialoprotein-binding protein | Adhesion to host extracellular matrix (ECM), intercellular aggregation, biofilm formation | Y | N |  |
|  | *Sbi* | IgG-binding protein SBI | Binds IgG and complement to block opsonization | Y | N |  |
|  | *scn* | Staphylococcal complement inhibitor | Inhibits the human complement system, a major part of innate immunity. | Y | N |  |
| **Iron-associated** | *isdA-I* | iron-regulated surface determinant protein A-I | Blood survival, abscess formation | Y | Y | EB05_00737- EB05_00741 |
|  | *srtB* | NPQTN specific sortase B | Required for heme utilization | Y | Y | EB05_00735" |

Genomic loci for *S. capitis* EB05 are shown as corresponding PROKKA-annotated nodes from the GenBank file. Due to limited database references for coagulase negative Staphylococcus (CoNS), functional prediction was based on homology and annotation tools. Y: yes (presence), N: No (absence).

**References**

1. Dadashi M, Hajikhani B, Darban-Sarokhalil D, van Belkum A, Goudarzi M. Mupirocin resistance in Staphylococcus aureus: A systematic review and meta-analysis. Journal of global antimicrobial resistance. 2020;20:238-47.

2. de Sena Brandine G, Smith AD. Falco: high-speed FastQC emulation for quality control of sequencing data. F1000Research. 2021;8:1874.

3. Quijada NM, Rodríguez-Lázaro D, Eiros JM, Hernandez M. TORMES: an automated pipeline for whole bacterial genome analysis. Bioinformatics. 2019;35(21):4207-12.

4. Schmieder R, Edwards R. Quality control and preprocessing of metagenomic datasets. Bioinformatics. 2011;27(6):863-4.

5. Bankevich A, Nurk S, Antipov D, Gurevich AA, Dvorkin M, Kulikov AS, et al. SPAdes: a new genome assembly algorithm and its applications to single-cell sequencing. Journal of computational biology. 2012;19(5):455-77.

6. Seemann T. Prokka: rapid prokaryotic genome annotation. Bioinformatics. 2014;30(14):2068-9.

7. Zankari E, Hasman H, Cosentino S, Vestergaard M, Rasmussen S, Lund O, et al. Identification of acquired antimicrobial resistance genes. Journal of antimicrobial chemotherapy. 2012;67(11):2640-4.

8. McArthur AG, Waglechner N, Nizam F, Yan A, Azad MA, Baylay AJ, et al. The comprehensive antibiotic resistance database. Antimicrobial agents and chemotherapy. 2013;57(7):3348-57.

9. Gupta SK, Padmanabhan BR, Diene SM, Lopez-Rojas R, Kempf M, Landraud L, et al. ARG-ANNOT, a new bioinformatic tool to discover antibiotic resistance genes in bacterial genomes. Antimicrobial agents and chemotherapy. 2014;58(1):212-20.

10. Chen L, Yang J, Yu J, Yao Z, Sun L, Shen Y, et al. VFDB: a reference database for bacterial virulence factors. Nucleic acids research. 2005;33(suppl_1):D325-D8.

11. Carattoli A, Zankari E, Garcìa-Fernandez A, Larsen MV, Lund O, Villa L, et al. PlasmidFinder and pMLST: in silico detection and typing of plasmids. Antimicrob Agents Chemother. 2014;58(7):3895-903.

12. Carattoli A, Zankari E, Garcia-Fernandez A, Voldby Larsen M, Lund O, Villa L, et al. In silico detection and typing of plasmids using PlasmidFinder and plasmid multilocus sequence typing. Antimicrob Agents Chemother. 2014;58(7):3895-903.

13. Jolley KA, Bray JE, Maiden MCJ. Open-access bacterial population genomics: BIGSdb software, the PubMLST.org website and their applications. Wellcome Open Res. 2018;3:124.

14. Johnson JS, Spakowicz DJ, Hong B-Y, Petersen LM, Demkowicz P, Chen L, et al. Evaluation of 16S rRNA gene sequencing for species and strain-level microbiome analysis. Nature communications. 2019;10(1):5029.

15. Jiao X, Zheng X, Ma L, Kutty G, Gogineni E, Sun Q, et al. A benchmark study on error assessment and quality control of ccs reads derived from the PacBio RS. J Data Mining Genomics Proteomics 4: 16008. 2013.

16. Martin M. Cutadapt removes adapter sequences from high-throughput sequencing reads. EMBnet journal. 2011;17(1):10-2.

17. Rognes T, Flouri T, Nichols B, Quince C, Mahé F. VSEARCH: a versatile open source tool for metagenomics. PeerJ. 2016;4:e2584.

18. Sierra MA, Li Q, Pushalkar S, Paul B, Sandoval TA, Kamer AR, et al. The influences of bioinformatics tools and reference databases in analyzing the human oral microbial community. Genes. 2020;11(8):878.

19. Parks DH, Chuvochina M, Chaumeil P-A, Rinke C, Mussig AJ, Hugenholtz P. A complete domain-to-species taxonomy for Bacteria and Archaea. Nature biotechnology. 2020;38(9):1079-86.

20. O'Leary NA, Wright MW, Brister JR, Ciufo S, Haddad D, McVeigh R, et al. Reference sequence (RefSeq) database at NCBI: current status, taxonomic expansion, and functional annotation. Nucleic acids research. 2016;44(D1):D733-D45.

21. Callahan BJ, McMurdie PJ, Rosen MJ, Han AW, Johnson AJA, Holmes SP. DADA2: High-resolution sample inference from Illumina amplicon data. Nature methods. 2016;13(7):581-3.

22. McMurdie PJ, Holmes S. phyloseq: an R package for reproducible interactive analysis and graphics of microbiome census data. PloS one. 2013;8(4):e61217.

23. Shannon CE. A mathematical theory of communication. The Bell system technical journal. 1948;27(3):379-423.

24. Oksanen J. Vegan: community ecology package. R package version. 2015;2:3.

25. Callahan BJ, Wong J, Heiner C, Oh S, Theriot CM, Gulati AS, et al. High-throughput amplicon sequencing of the full-length 16S rRNA gene with single-nucleotide resolution. Nucleic acids research. 2019;47(18):e103-e.
